# Synergistic Regulation at Physiological, Transcriptional, and Metabolic Levels in *Dendrobium huoshanense* Plants Under Combined Drought and High-Temperature Stress

**DOI:** 10.3390/genes16030287

**Published:** 2025-02-27

**Authors:** Xingen Zhang, Guohui Li, Peipei Wei, Binbin Du, Shifan Liu, Jun Dai

**Affiliations:** 1 Generic Technology Research Center for Anhui Traditional Chinese Medicine Industry, West Anhui University, Lu’an 237012, China; xingenzhang2023@163.com; 2 College of Biotechnology and Pharmaceutical Engineering, West Anhui University, Lu’an 237012, China; guohuili23112@163.com (G.L.); peipeiw23778@163.com (P.W.); dubinbin2233@163.com (B.D.); liushifan0321@163.com (S.L.)

**Keywords:** *Dendrobium huoshanense*, combined drought–high-temperature stress, transcriptome, metabolome, synergistic regulation

## Abstract

**Background:** With global warming and climate change, the occurrence of abiotic stresses has become increasingly prevalent. Drought often occurs with high temperatures, especially in arid and semi-arid regions. However, the molecular mechanisms of plants responding to combined drought and high-temperature stress remains unclear. **Results:** Through integrative physiological, transcriptomic, and metabolomic analyses, we systematically investigated the adaptive mechanisms of *Dendrobium huoshanense* under combined drought and high-temperature stress. Our findings revealed that combined drought and high-temperature stress led to significant reductions in photosynthetic efficiency and increased oxidative damage in *Dendrobium huoshanense*, with high-temperature stress being the primary contributor to these adverse effects. The joint analysis shows that three core pathways—signal transduction, lipid metabolism, and secondary metabolite biosynthesis—were identified as critical for antioxidant defense and stress adaptation. **Conclusions:** These findings not only deepen our understanding of plant responses to combined drought and high-temperature stress but also provide new directions for future research on the cultivation and resistance improvement of *Dendrobium huoshanense*.

## 1. Introduction

In recent years, with the intensification of global climate change, high-temperature and drought stresses often occur simultaneously or sequentially, creating complex combined stresses. Their impact on agricultural production systems and socio-economic structures far exceeds that of single high-temperature or drought stress. Compared to single stresses, the combined stress of drought and high temperature often results in synergistic effects, with greater harm [1]. Therefore, it is crucial to analyze plant responses to combined stress in order to enhance resilience and develop more effective mitigation strategies.

The combined drought and high-temperature stress has a complex synergistic effect on plant physiological ecology, with photosynthesis being the primary target under these stress conditions. High temperature not only significantly decreases the CO_2_ affinity of Rubisco, a key enzyme in photosynthesis [2], but also inactivates Rubisco through thermal damage, leading to a significant reduction in the efficiency of net photosynthesis [3]. Drought conditions further exacerbate the situation by inhibiting the diffusion of CO_2_ through stomata and chloroplasts, directly affecting carbon fixation [4]. In plants, the reaction centers of PSI and PSII are major sites for the production of reactive oxygen species (ROS), generating superoxide (O_2_^−^) and singlet oxygen (^1^O_2_), respectively, under excess light [5]. Furthermore, photorespiration, which is enhanced under high temperature, promotes ROS production by generating H_2_O_2_ in the peroxisome [6]. Given that both drought and high temperature can induce ROS production through similar mechanisms, the combined stresses result in impaired carbon fixation from drought and disrupted photorespiration from high temperature, leading to a severe imbalance in redox homeostasis and exacerbating ROS accumulation [7]. The excessive accumulation of ROS is particularly harmful to the photosynthetic machinery, including the oxidation of membrane lipids and proteins [8]. These reactive species not only damage cellular structures such as membranes, proteins, and nucleic acids but also activate programmed cell death pathways, ultimately leading to cell death [9]. In this context, carotenoids play a crucial role in photoprotection by scavenging ROS and quenching excited singlet states of chlorophyll [10]. Carotenoids, as antioxidants, neutralize ROS, particularly singlet oxygen (^1^O_2_) and peroxyl radicals, preventing oxidative damage to cellular structures. Additionally, carotenoids help protect the photosynthetic machinery by quenching the excited singlet state of chlorophyll. When chlorophyll absorbs light and enters the excited singlet state, this energy can either be transferred to the reaction center to drive photosynthesis or decay into a triplet state, which can then react with molecular oxygen to produce ROS [10,11]. Carotenoids intercept this energy, rapidly converting the excited singlet state of chlorophyll into heat, thereby dissipating excess energy and preventing it from transferring to the reaction center and generating ROS [10,11]. This “quenching” mechanism is vital for protecting the photosynthetic apparatus and alleviating oxidative damage under stress conditions.

To cope with stress, plants have developed a number of highly efficient and sophisticated strategies during their long biological evolution. First, they activate the ROS scavenging mechanism, which centers on the synergistic action of a series of enzymes (e.g., superoxide dismutase, catalase, ascorbate peroxidase, and glutathione peroxidase) and antioxidants (e.g., ascorbate and glutathione) that are significantly increased in response to adversity and are effective in counteracting oxidative stress [11]. Moreover, the combined stress of drought and high temperature can induce significant alterations in plant metabolic profiles, resulting in the production of a substantial amount of secondary metabolites. The secondary metabolites, as an important part of the plant defense system, mitigate high-temperature injury by reducing polyunsaturated fatty acid synthesis and decreasing cell membrane fluidity at high temperatures [12]. For example, under drought conditions, specific secondary metabolites such as flavonoid glycosides exhibit antioxidant properties that enhance drought tolerance in plants such as red clover [13]. At the same time, plants synthesize a variety of compatible solutes in response to abiotic stress. For example, the sugar family oligosaccharides (RFOs) of cotton accumulate significantly during high-temperature stress compared to drought, providing osmotic protection [14,15]. Previous studies have revealed detailed signaling networks under single drought and high-temperature stress conditions. However, how the combined action of drought and high-temperature stress affects specific signaling pathways remains largely unknown. Using histological techniques, the molecular mechanisms of some plants in response to drought and high-temperature stress have been revealed. For example, in maize, Yang et al. analyzed the transcriptome of the primary root under drought–high-temperature stress and found that GOs associated with DNA-binding factors were enriched in combined stress-specific up-and-down-regulated genes [16]. In *Arabidopsis*, proteomic analysis revealed that *ASCORBATE PEROXISOME 1* (*APX1*) plays an important role in response to combined stress [17].

*Dendrobium huoshanense* (*D. huoshanense*), a prized traditional Chinese medicinal herb, is renowned for its notable effects in nourishing Yin, tonifying the stomach, clearing heat, detoxifying, and relieving coughs. It has a long history of use in traditional Chinese medicine and is hailed as the “King of Herbs”. Its unique growing environment makes it highly susceptible to the impacts of drought and high temperatures. Here, we subjected *D. huoshanense* to drought, high-temperature, and drought–high-temperature composite stresses and conducted a synergistic analysis of the resulting data using physiological, transcriptomic, and metabolomic approaches. This study aims to investigate the physiological responses of *D. huoshanense* to combined drought and high-temperature stress, and the results demonstrated that signal transduction, lipid metabolism, and secondary metabolite biosynthesis pathways played a pivotal role in response to combined stress. By elucidating these pathways, we seek to provide a theoretical basis for future genetic improvement and cultivation practices aimed at enhancing the plant’s stress resilience.

## 2. Materials and Methods

### 2.1. Plant Material and Treatment

Three-month-old *D. huoshanense* tissue culture seedlings were grown in growth chambers under a 12 h/25 °C day and 12 h/22 °C night regimen, with 70% relative humidity and white light with a light intensity of 150 µmol·m^−2^·s^−1^. Uniformly grown, robust seedlings were selected for treatment. To simulate drought and high-temperature stresses, seedlings were treated with 20% PEG6000 and exposed 42 °C, respectively. For combined stress, plants were transferred to an MS medium containing 20% PEG6000 and incubated at 42 °C.

### 2.2. Measurement of Physiological Indicators

Leaves and stalks of *D. huoshanense* were collected at 0, 24, 48, and 72 h after treatment for physiological measurements. MDA content and SOD activity were measured using kits according to the manufacturer’s instructions (Jiangsu Bioisco Biotechnology, LLC, Nanjing, China). POD and CAT activities were measured using kits following the manufacturer’s protocols [5,13] (Nanjing Jiancheng Bioengineering Institute, Nanjing, China).

### 2.3. Transcriptome Sequencing and Data Analysis

Total RNA was extracted from each sample using the TRIzol Reagent Mini Kit (Qiagen, Hilden, Germany). RNA quantity and quality were assessed using the Agilent 2100 Bioanalyzer (Agilent Technologies, Palo Alto, CA, USA) and NanoDrop (Thermo Fisher Scientific Inc., Waltham, MA, USA). RNA samples with integrity numbers (RIN) above 9 (1 μg) were used for library construction. Poly(A) mRNA was isolated using the NEBNext^®^ Poly(A) mRNA Magnetic Isolation Module (New England Biolabs Inc., Beverly, MA, USA), and cDNA libraries were prepared using the NEBNext^®^ Ultra™ RNA Library Prep Kit for Illumina^®^ (New England Biolabs Inc., Beverly, MA, USA). Libraries were multiplexed with different indices and loaded on an Illumina HiSeq platform (Illumina, San Diego, CA, USA) according to the manufacturer’s instructions.

Raw reads were processed using FASTQC (v.0.11.9) to obtain clean reads. Clean reads were mapped to the *D. huoshanense* genome using STAR (v2.7.9a). FPKM (fragments per kilobase of transcript per million fragments mapped) values for each gene were calculated, and read counts were obtained using RSEM (v1.3.3). Principal component analysis (PCA) was performed using the “gmodels” package in R v 3.2.0 (Ihaka and Gentleman, New Zealand, 1996). Differentially expressed genes (DEGs) were identified based on the following parameters: fold change ≥ 2.00, probability ≥ 0.8, and false discovery rate (FDR) < 0.05, with three biological replicates. Gene Ontology (GO) and Kyoto Encyclopedia of Genes and Genomes (KEGG) enrichment analyses of DEGs were performed using the clusterProfiler package (version 3.8).

### 2.4. Metabolite Extraction, Quantification, and Metabolomics Analysis

A 100 μL sample was mixed with 400 μL of extraction solution (MeOH: ACN, 1:1 *v*/*v*) containing deuterated internal standards. The mixture was vortexed for 30 s, sonicated for 10 min in a 4 °C water bath, and incubated at −40 °C for 1 h to precipitate proteins. The samples were then centrifuged at 12,000 rpm (RCF = 13,800× *g*) for 15 min at 4 °C. The supernatant was transferred to a fresh glass vial for analysis. A quality control (QC) sample was prepared by pooling equal aliquots of the supernatant from all samples.

LC-MS/MS analysis was performed using a Vanquish UHPLC system (Thermo Fisher Scientific Inc., Waltham, MA, USA) coupled with a Waters ACQUITY UPLC BEH Amide column (2.1 mm × 50 mm, 1.7 μm) and Orbitrap Exploris 120 mass spectrometer (Thermo). The mobile phase consisted of 25 mmol/L ammonium acetate and 25 mmol/L ammonia hydroxide in water (pH = 9.75) (A) and acetonitrile (B). The autosampler was set at 4 °C, with an injection volume of 2 μL. The mass spectrometer was operated in information-dependent acquisition (IDA) mode. ESI source conditions included sheath gas flow at 50 Arb, auxiliary gas flow at 15 Arb, capillary temperature at 320 °C, full MS resolution at 60,000, MS/MS resolution at 15,000, collision energy (SNCE) at 20/30/40, and spray voltage at 3.8 kV (positive) or −3.4 kV (negative).

Raw data were converted to mzXML format using ProteoWizard and processed with an in-house program based on XCMS for peak detection, extraction, alignment, and integration. Differential metabolites were identified and quantitatively analyzed using the MetaX software (version 3.x). Screening criteria included fold change ≥ 2 (absolute value of log2 FC ≥ 1), false discovery rate (FDR) < 0.05, and variable importance for projection (VIP) value ≥ 1.

### 2.5. Combined Transcriptome and Metabolome Analysis

Combined transcriptomic and metabolomic analyses were performed based on KEGG metabolic pathways. The “C vs. DHT” comparison group was used for analysis. DEGs were identified, and their KEGG enrichment was analyzed to identify key pathways. Metabolites that accumulated differently between the stress treatments were also analyzed. KEGG enrichment analysis of differential metabolites revealed important pathways. To clarify key KEGG pathways in *D. huoshanense* in response to drought–high-temperature stress, transcriptomics and metabolomics data were cross-analyzed. Comparative analysis of the “D group vs. DHT group” and “HT group vs. DHT group” was also conducted to identify key metabolic pathways containing significant genes. The network of relationships between key pathways, differentially expressed genes, and metabolites was visualized using ChiPlot (https://www.chiplot.online/) (accessed on 9 May 2024).

### 2.6. Data Analysis

Physiological data were analyzed using analysis of variance (ANOVA) with IBM SPSS Statistics 16.0 (SPSS Inc., Chicago, IL, USA), with significance set at *p* < 0.05 for three treatment groups.

## 3. Results

### 3.1. Biochemical and Physiological Changes of D. huoshanense Under Single and Compound Stress

**Figure 1:**
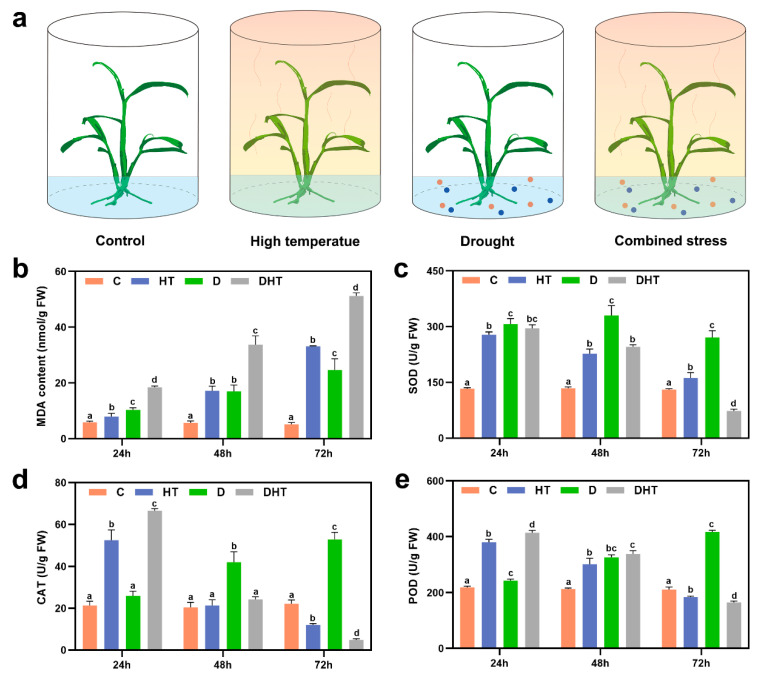
Physiological responses of *D. huoshanense* to drought, high-temperature, and combined stresses. (**a**) Schematic diagram of the experimental design. Colored dots represent a schematic diagram of drought stress. (**b**–**e**) Effects of different stress types on total malondialdehyde (MDA) content, superoxide dismutase (SOD) activity, catalase (CAT) activity, and peroxidase (POD) activity. Data are presented as mean ± standard deviation (SD). One-way ANOVA was used to compare groups, with post-hoc Tukey’s test to determine pairwise differences. *p* < 0.05 was considered statistically significant. Bars with different letters indicate statistically significant differences between groups (*p* < 0.05).

To determine the critical time points for transcriptome and metabolome sampling, we examined changes in physiological indicators at various time points during the treatment. Under drought stress, the MDA content and the activities of SOD, POD, and CAT showed an increasing trend as the treatment duration extended (Figure 1). In contrast, under high-temperature stress, the MDA content continued to rise, while POD, SOD, and CAT activities peaked at 24 h and then decreased (Figure 1). For combined stress, the trends observed in MDA, POD, SOD, and CAT followed those under high-temperature stress, but the magnitude of these changes was more pronounced (Figure 1). These findings indicate that prolonged exposure to high temperatures adversely impacts the metabolic activities in *D. huoshanense*, with combined stress causing more significant damage than either stress alone.

### 3.2. Analysis of Transcriptome Sequencing Results and Functional Annotation of Differentially Expressed Genes

**Figure 2:**
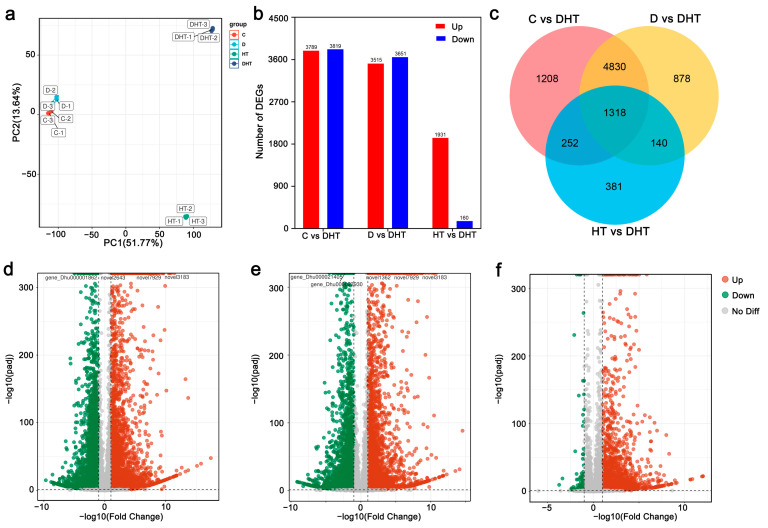
Distribution of differentially expressed genes in *D. huoshanense* under various stress conditions. (**a**) Principal component analysis (PCA) of the transcriptome of *D. huoshanense*. (**b**) The number of genes that were up-regulated (left box) or down-regulated (right box) in plants subjected to different stressors. (**c**) Venn diagram showing the overlap of DEGs in plants under different stress conditions. (**d**–**f**) Volcano plots of up-regulated and down-regulated DEGs in plants under C vs. DHT and D vs. DHT stress conditions, respectively.

Physiological indicators confirmed that the combined drought–high-temperature stress had a greater impact on *D. huoshanense* than single stresses. To explore the molecular mechanisms of this effect, the transcriptome of *D. huoshanense* under the three treatments was sequenced and analyzed. PCA results showed distinct separation of the four treatment groups in the PC1 × PC2 score plot, with a noticeable grouping between the DHT and HT groups (Figure 2a). Transcriptome analysis identified DEGs using a threshold of *p* < 0.05 and Log2FC ≥ 1. In comparison to DHT, 7608 (3819 up-regulated, 3789 down-regulated), 7166 (3651 up-regulated, 3515 down-regulated), and 2091 (160 up-regulated, 1931 down-regulated) DEGs were identified in the C, D, and HT treatments, respectively (Figure 2b,d–f). A comparison of the three groups (C vs. DHT, HT vs. DHT, and D vs. DHT) revealed 1318 common DEGs (Figure 2c), which are likely important in the plant’s response to combined stress.

**Figure 3:**
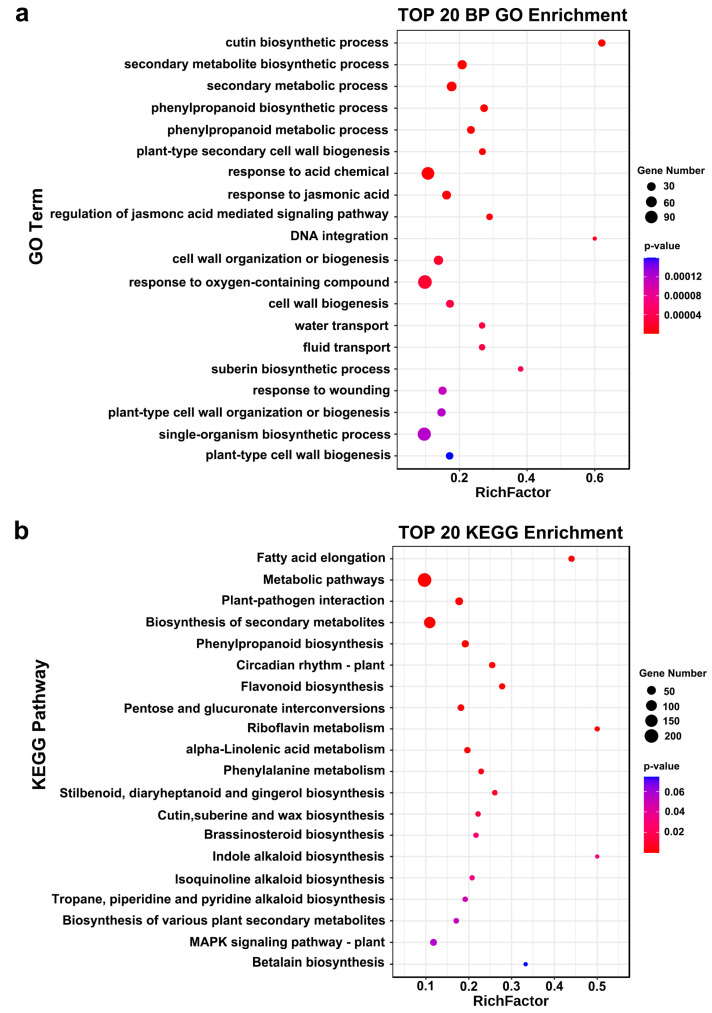
Gene Ontology (GO) and Kyoto Encyclopedia of Genes and Genomes (KEGG) pathway enrichment analysis of the hub DEGs. (**a**,**b**) The top 20 GO biological processes and KEGG pathways enriched in the DEGs shared by C vs. DHT, D vs. DHT, and HT vs. DHT are presented. The *x*-axis represents the enrichment factor, and the *y*-axis represents the pathway name. The size of the bubbles reflects the number of DEGs involved, while the color indicates the degree of pathway enrichment.

GO enrichment analysis categorized these DEGs into three major categories: cellular components, molecular functions, and biological processes. For cellular components, DEGs were enriched in the “cell membrane” and “cell wall”. In molecular functions, DEGs were enriched in “monooxygenase activity”, “oxidoreductase activity”, “UDP-glycosyltransferase activity”, and “transferase activity, transferring glycosyl groups” (Table S1). Biological processes were primarily enriched in “response to oxygen-containing compounds”, “response to jasmonic acid”, and “secondary metabolic processes” (Figure 3a). KEGG enrichment analysis showed significant enrichment of DEGs in “metabolic pathways”, “biosynthesis of secondary metabolites”, and “flavonoid biosynthesis” (Figure 3b).

### 3.3. Analysis of Metabolome Sequencing Results and Functional Annotation of Differential Metabolites (DEM)

**Figure 4:**
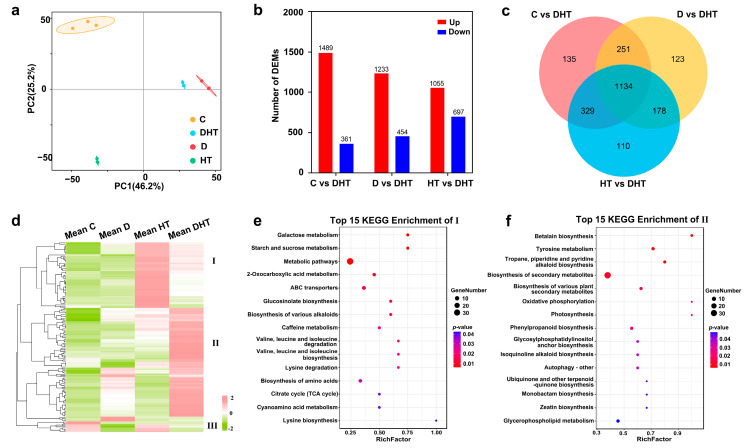
The distribution of differential metabolites in *D. huoshanense* under drought, high-temperature, and combined drought–high-temperature stress. (**a**) Principal component analysis (PCA) of the metabolome in *D. huoshanense*. (**b**) Statistical plots of DEMs in plants subjected to different stressors. (**c**) Venn diagram of DEMs in plants under various stresses. (**d**) Cluster analysis of differential metabolites shared by the C vs. DHT, D vs. DHT, and HT vs. DHT groups. (**e**,**f**) KEGG enrichment analysis of class I and II differential metabolites from panel (**d**).

Further, we performed metabolomics as shown in Figure S2 TIC plots of UHPLC-OE-MS detection of positive (right) and negative (left) ion patterns of QC samples. PCA of the metabolomics data showed that replicate samples from both control and stress groups clustered tightly, indicating reproducibility of the data (Figure 4a). A total of 2321 metabolites were identified through secondary mass spectrometry. A comparison between the C vs. DHT, D vs. DHT, and HT vs. DHT groups identified 1850, 1687, and 1752 differential metabolites (DEMs), respectively (Figure 4b). These metabolites primarily included lipids and lipid-like molecules (15%), organoheterocyclic compounds (14%), organic acids and derivatives (9%), benzenoids (9%), organic oxygen compounds (5%), phenylpropanoids and polyketides (5%), and others (Figure S1a).

KEGG enrichment analysis of metabolites from the three comparison groups revealed that all groups were enriched in pathways related to phenylalanine, tyrosine, and tryptophan biosynthesis, phenylpropanoid biosynthesis, caffeine metabolism, citrate cycle (TCA cycle), nucleotide metabolism, amino acid biosynthesis, and ABC transporters (Figure S1b). Further analysis of the 1120 differential metabolites identified across all three groups indicated that 159 metabolites clustered into three major groups (Figure 4d). The first group, which showed the highest expression in the HT group, was primarily enriched in “metabolic pathways”, “starch and sucrose metabolism”, and “galactose metabolism” (Figure 4e). The second group, showing the highest expression in the DHT group, was enriched in “biosynthesis of secondary metabolites”, “tropane, piperidine, and pyridine alkaloid biosynthesis”, “betalain biosynthesis”, and “tyrosine metabolism” (Figure 4f).

### 3.4. Conjoint Analysis of Transcriptomics and Metabolomics

To better understand the molecular mechanisms of combined drought and high-temperature stress, we conducted a joint analysis of DEGs and DEMs from the C vs. DHT, D vs. DHT, and HT vs. DHT groups. The results revealed that the DHT group showed significant specificity in several biological processes compared to the other three groups (Table S2). Notably, differential genes and metabolites were enriched in phytohormone signaling and mitogen-activated protein kinase (MAPK) signaling pathways, suggesting extensive plant adaptations through these pathways under combined stress. Additionally, significant changes were observed in secondary metabolite synthesis pathways, particularly those involved in isoflavonoid biosynthesis, flavonoid biosynthesis, stilbenoid and diarylheptanoid biosynthesis, and phenylpropanoid biosynthesis. Lipid metabolic pathways, such as cutin, suberin, and wax biosynthesis, fatty acid elongation, and α-linolenic acid metabolism, were also significantly enriched across all groups (Table S2).

**Figure 5:**
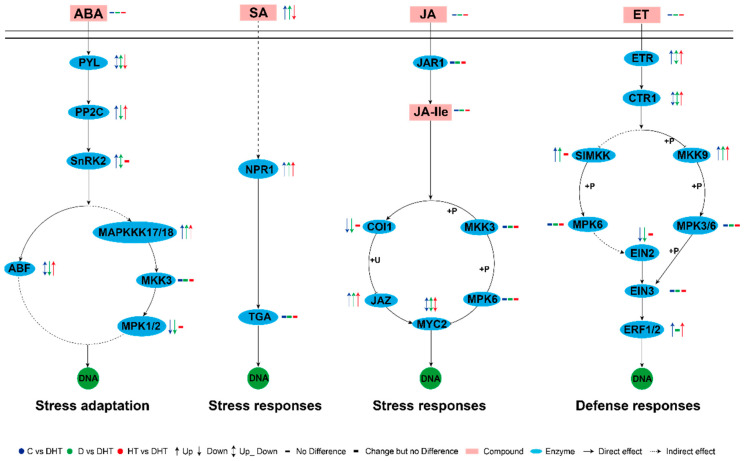
Reconstruction of some hormone signaling pathways and MAPK signaling pathways in *D. huoshanense* through joint analysis of structural genes and metabolites under stress conditions. The coding genes indicated by gene symbols are described as follows: ABA, abscisic acid; PYL, pyrabactin resistance 1-like; PP2C, protein phosphatase 2C; SnRK2, serine/threonine protein kinase 2; ABF, ABA-responsive element binding factor; MAPKK17/18, mitogen-activated protein kinase kinase 17/18; MKK3, mitogen-activated protein kinase kinase 3; MPK1/2, mitogen-activated protein kinase 1/2; SA, salicylic acid; NPR1, non-expressor of pathogenesis-related genes 1; TGA, basic leucine zipper transcription factor; JA, jasmonic acid; JA-Ile, jasmonoyl-L-isoleucine; COI1, coronatine-insensitive protein 1; JAZ, jasmonate ZIM domain-containing protein; MPK6, mitogen-activated protein kinase 6; MYC2, myelocytomatosis protein 2; ET, ethylene; ETR, ethylene receptor; CTR1, constitutive triple response 1; SIMKK, mitogen-activated protein kinase 4/5; EIN2, ethylene-insensitive protein 2; MKK9, mitogen-activated protein kinase kinase 9; EIN3, ethylene-insensitive protein 3; and ERF1/2, ethylene-responsive transcription factor 1/2.

**Figure 6:**
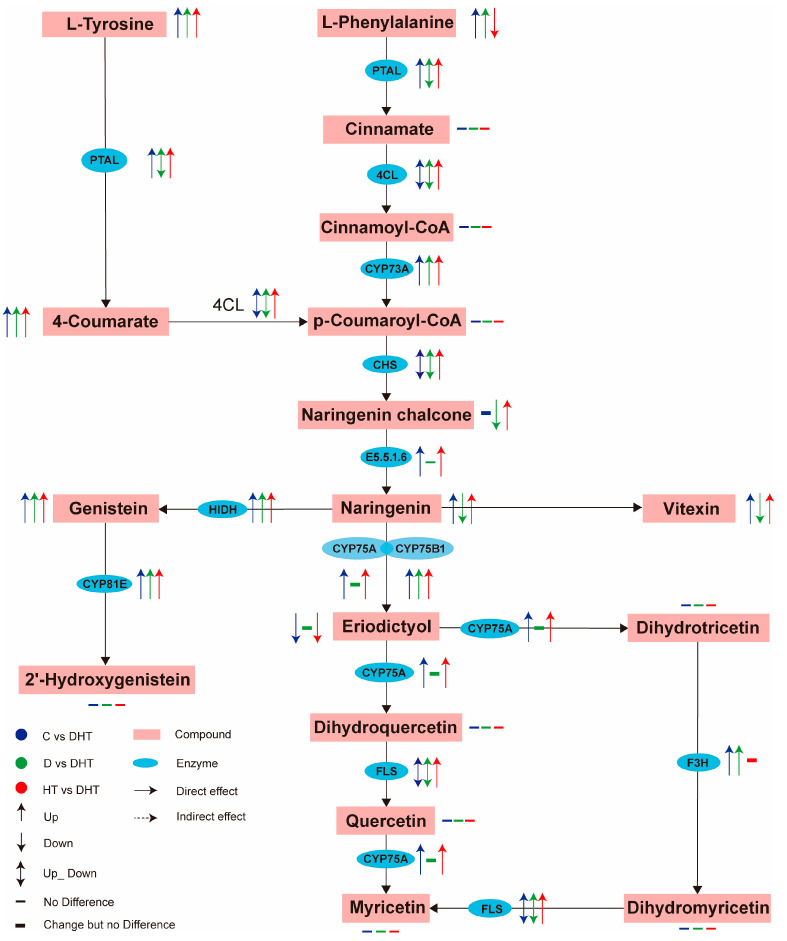
Reconstruction of the flavonoid synthesis pathway in *D. huoshanense* through joint analysis of structural genes and metabolites under stress conditions. The coding genes indicated by gene symbols are described as follows: PTAL, phenylalanine/tyrosine ammonia-lyase; 4CL, 4-coumarate-CoA ligase; CYP73A, trans-cinnamate 4-monooxygenase; CHS, chalcone synthase; E5.5.1, chalcone isomerase; HIDH, 2-hydroxyisoflavanone dehydratase; PAL, phenylalanine ammonia-lyase; C4H, cinnamate 4-hydroxylase; CYP75A, flavonoid 3′,5′-hydroxylase; CYP75B1, flavonoid 3′-monooxygenase; CYP81E, isoflavone/4′-methoxyisoflavanone 2′-hydroxylase; F3H, flavonoid 3-hydroxylase; and FLS, flavonol synthase.

At both the transcriptional and translational levels, pathways related to basal transcription factors, spliceosomes, mRNA surveillance, and ribosomes were enriched, reflecting the plant’s broad adaptability in regulating gene expression. Special attention was given to the signaling and flavonoid synthesis pathways. Overlap between the phytohormone and MAPK signaling pathways was evident, with key genes and enzymes showing coordinated up- and down-regulation across the treatment groups (Figure 5). In particular, the SA-NPR1 pathway, marked by significant changes in salicylic acid (SA) and NPR1 expression, played a central role in the plant’s signaling response. Additionally, changes in genes related to abscisic acid (ABA), jasmonic acid (JA), and ethylene (ET) signaling pathways highlighted the complex regulatory mechanisms of the phytohormone network. In flavonoid synthesis, we integrated data from compound and gene signaling networks using the KEGG database, showing enhanced metabolic activity in phenylpropanoid biosynthesis, which serves as a precursor for flavonoid synthesis. Key enzymes and genes involved in isoflavonoid and flavonoid biosynthesis were identified, revealing the fine regulation of secondary metabolites in response to stress (Figure 6).

## 4. Discussion

### 4.1. High-Temperature Stress Can Be Considered the Dominant Factor When Combined Drought and High-Temperature Stress Occurs

The interconnection of genes and phenotypes is an important way to address scientific questions [18,19]. The joint analysis of transcription and metabolism can also provide us with a more accurate understanding of plant life activities [20]. Compound stress can be considered as a new type of stress due to the interactions between stressors [7,21]. Phenotypic and physiological changes in plants can be unique when faced with multifactorial stress combinations [22]. In general, plants tend to exhibit more severe phenotypic changes under combined stresses than under single stresses [23,24]. Zandalinas, Sengupta et al. [25] demonstrated using low levels of stress that although each individual stress had negligible effects on the plant, multifactorial stress combinations had deleterious cumulative effects on *Arabidopsis*. In contrast, Rivero et al. (2014) showed that a combination of salt–high-temperature stress had a protective effect on *D. huoshanense* compared to a single stress [26]. Here, we suggest that the interaction between drought and high-temperature stress ultimately affects the genes, metabolites, physiological responses, and growth of *D. huoshanense* under combined drought and high-temperature stress, which may depend on the extent of each stress level involved in the stress combination, as well as the genotype of the plant. Zhou’s study found that drought was a major stressor for tomato plants under combined drought and high-temperature stress [24,27]. In this study, we found that *D. huoshanense* plants under combined stress exhibited different characteristics at the growth, physiological, transcriptional, and metabolic levels than those of the single stress. For example, in terms of leaf defense enzyme activities such as POD and other physiological indices, *D. huoshanense* plants under combined stress showed more similar characteristics (Figure 1). In addition, PCA component analysis of both transcriptome and metabolome showed that groups C and D were clustered in closer positions, while groups HT and DHT were more distant (Figure 2a and Figure 4a). Based on the above events, we suggest that high-temperature stress may play a dominant role in the plant response to combined stress.

### 4.2. Signaling in D. huoshanense Under Combined Drought and High-Temperature Stress

In the context of combined stressors, the enrichment of hormone signaling pathways and MAPK cascades was notable, with these pathways displaying potential detoxification and adaptation mechanisms [28,29,30,31]. In this study, the expression pattern of ABA receptors pyrabactin resistance 1-like (PYL) and protein phosphatase 2C (PP2C) was found to be significantly altered in the DHT group, when compared to the control group C, the drought group D, and the high-temperature group HT. The PYL, an ABA-specific receptor, was observed to bind and inhibit the activity of PP2C in the presence of ABA, thereby derepressing Sucrose Non-fermenting 1-Related Protein Kinase 2 (SnRK2) [32,33,34]. This resulted in the rapid activation of ABF (ABA-Responsive Element Binding Factors) and MAPKKK17/18 genes, which in turn triggered a response to drought and high-temperature stress [35,36,37,38]. In contrast, the concentration of salicylic acid (SA) in the DHT group was markedly distinct from that of the other three groups. Additionally, the expression of the SA receptor nonexpresser of pathogenesis-related genes 1 (NPR1) exhibited an upward trajectory, not only triggering the expression of the NPR1 gene but also facilitating a conformational change of NPR1, which was translocated from the cytoplasm to the nucleus; the accumulation of NPR1 in the nucleus can activate the expression of a series of defense genes, thus enhancing plant stress tolerance [39,40,41]. Furthermore, the expression of the key target genes MYC2 and ERF1/2 (Ethylene Responsive Factors 1/2) in the JA and ET signaling pathways in the DHT group differed from that observed in the other three groups [42,43,44]. These two genes were also demonstrated to be capable of directly activating the defenses of *D. huoshanense* in response to stress. The two genes in question are capable of directly activating the defense system of *D. huoshanense*, thereby enabling the plant to respond to stress. It is noteworthy that the chitinase B (*CHiB*) and vegetative storage protein (*VP*) genes, which act as the primary regulators of the defense response in plants, were simultaneously regulated by *MYC2* and *ERF1/2* within the MAPK signaling cascade pathway [45,46,47,48,49]. In the DHT group, the expression of the *CHiB* and *VP* gene exhibited a tendency towards increased regulation in comparison to the other three groups. This suggests that *D. huoshanense* initiated a more robust defense response to the challenging circumstances presented by stress through the up-regulation of the expression of the *CHiB* and *VP* genes in the context of drought and high-temperature composite stress conditions.

### 4.3. Oxidative and Antioxidant Mechanisms of D. huoshanense Under Combined High-Temperature and Drought Stress

In conditions of elevated stress, the metabolic pathways of plants exert a pronounced influence on the regulation of their antioxidant defense mechanisms. The present study concentrated on the physiological responses of *D. huoshanense* when subjected to single versus compound stress conditions. The results demonstrated that the combined stressor resulted in a notable elevation in malondialdehyde (MDA) content in *D. huoshanense* when compared to the single stressor (Figure 1b). This finding suggests that the combined stressor triggered a heightened level of free radical generation and a more intense membrane lipid peroxidation process. Concomitantly, the activities of peroxidase (POD), superoxide dismutase (SOD), and catalase (CAT) were enhanced, with a particularly pronounced elevation of POD activity, in contrast to the control (Figure 1c–e). It is noteworthy that the expression of key genes regulating the activities of these enzymes also exhibited significant alterations. These include genes related to POD, SOD, and CAT (Table S3). These observations collectively substantiate the proposition that the combined stressor results in a substantial accumulation of reactive oxygen species (ROS) within the plant. It is postulated that the ROS signals may act as triggers to activate the expression of related enzymes and genes, which in turn exert a synergistic regulatory influence on the adaptive responses of the plant to combined stress.

**Figure 7:**
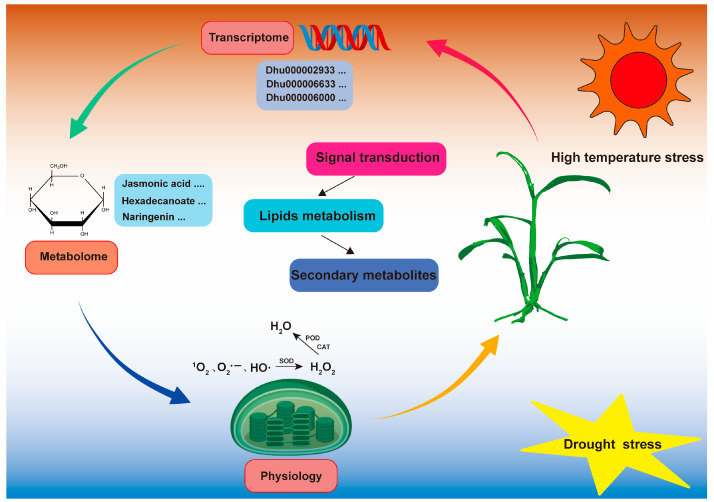
Regulatory mechanism in *D*. *huoshanense* responding to the combination of drought and high-temperature stress. The three key pathways are shown in different colors in the lower left corner. POD, peroxidase; CAT, catalase; SOD, superoxide dismutase; ^1^O_2_, O_2_−, HO, reactive oxygen species.

The role of fatty acids in plant response to stress cannot be overlooked, particularly given their involvement in crucial processes such as cytokinesis, signaling, activation of mitochondrial function, and the mitigation of membrane lipid peroxidation [50,51,52]. In response to drought stress, phospholipid molecules undergo degradation, resulting in the generation of phosphatidic acid (PA). As a pivotal second messenger, PA restricts intermembrane transfer and function by binding to target proteins [53,54]. For example, PA binds to phosphatases in the ABA signaling pathway, impeding its movement towards the nucleus and, as a consequence, attenuating the negative regulation of ABA and promoting stomatal closure in response to stress [53,54]. This study revealed significant alterations in the lipid metabolism of *D. huoshanense* in response to combined stress, as indicated by the variations in the expression and activity of acetyl coenzyme A acyltransferase 2 (ACAA2, EC:2). Significant differences in expression or activity were observed for 3.1.16, mitochondrial enoyl [acyl carrier protein] reductase (MECR, EC:1.3.1.38), and palmitoyl protein thioesterase (EC:3.1.2.22) compared to the control or other stress groups (Figure 4, Figure 6 and Figure 7). In particular, *ACAA2* was involved in the β-oxidation of fatty acids [55,56], whereas MECR acted on enoyl coenzyme A derivatives with carbon chain lengths ranging from 4 to 16. In response to stress conditions, *D. huoshanense* may optimize the unsaturated fatty acid content by regulating the expression levels of *ACAA2* and *MECR*. This not only helps to reduce lipid peroxidation but also generates signaling molecules to promote growth and alleviate stress toxicity. This reflects the fine metabolic regulation strategy of plants in complex stress environments.

Secondary metabolites are of great importance in the antioxidant mechanisms of plants. The present study demonstrated that DEGs and DEMs were markedly enriched in phenylpropanoid, flavonoid, and isoflavonoid synthesis pathways in the combined stress when compared to the control group C, drought group D, and high-temperature group HT. Flavonoids, a class of secondary metabolites with notable antioxidant capacity, have the potential to mitigate the adverse effects of drought by effectively resisting oxidative damage when plants are subjected to drought stress [57,58]. The antioxidant properties of flavonoids are primarily ascribed to the catechol moiety present on their B ring, wherein the hydroxyl group is capable of directly binding to and quenching ROS, leading to the formation of stable products [59,60]. It has been demonstrated that the regulation of functional genes associated with flavonoid biosynthesis can influence the flavonoid content of plants, which in turn affects their drought tolerance [61,62]. To illustrate, the production of flavonol glycoside 3 (*PFG3*), *MYB12*, and *MYB75* represent pivotal transcription factors that regulate flavonoid biosynthesis. In *Arabidopsis*, the loss of function of *PFG3* results in the blockage of flavonoid biosynthesis, thereby rendering the plants more sensitive to drought stress [62]. Conversely, the overexpression of *MYB12* and/or *MYB75* has been observed to increase flavonoid content in *Arabidopsis* and reduce the accumulation of ROS under drought stress, thereby enhancing the plant’s drought tolerance [61].

In the synthesis pathway of flavonoids, phenylalanine is initially catalytically converted to cinnamic acid by phenylalanine ammonia-lyase (*PAL*). This is then hydroxylated by cinnamic acid 4-hydroxylase (*C4H*) to produce coumaric acid. Coumaric acid is then catalyzed by 4-coumarate coenzyme A ligase (*4CL*) to form coumaroyl coenzyme A (CoA). Chalcone synthase (*CHS*) catalyzes the synthesis of chalcone from coumaroyl CoA and malonyl CoA derived from acetyl CoA. Subsequently, chalcone isomerase (*CHI*) catalyzes the isomerization of chalcone to colorless naringenin. Naringenin then enters the synthesis pathway of other flavonoids. The present study revealed that the expression of numerous flavonoid synthesis precursors and pivotal genes underwent notable alterations in the DHT group when compared to the remaining three groups. Specifically, the synthesis of flavonoids involves the conversion of various precursors, including phenylalanine and L-tyrosine to P-coumaric acid, chalcone, and naringenin. This process also leads to the formation of compounds such as vitexin and genistein. Additionally, the expression of genes encoding key enzymes in this pathway, such as *4CL*, *CHS*, *FLS* (flavonol synthetase), *CYP75* (a member of the cytochrome P450 family involved in flavonoid metabolism), and *F3H* (flavanone 3-hydroxylase), also undergoes significant changes. Significant alterations in gene expression were observed for hydroxylase and other genes. These metabolites and genes play a significant role in flavonoid metabolism and the response to environmental stress. For example, in *Arabidopsis CHS* gene mutants, the inability of leaf defense cells to synthesize flavonols properly resulted in a reduction in drought tolerance in *Arabidopsis* [63]. In citrus, the cytochrome P450 gene *CsCYT75B1* is involved in the accumulation of antioxidant flavonoids and induces drought resistance in transgenic *Arabidopsis* [58].

## 5. Conclusions

By integrating phenotypic observations, transcriptomic analyses, and metabolomic data, our study provides valuable insights into the adaptive resilience mechanisms of D. huoshanense under the combined stresses of drought and high temperature. Compared to individual stresses, the combined effects of these two stressors elicited a significantly stronger physiological response, with high-temperature stress emerging as the dominant factor. Through the analysis of DEGs and DEMs, we identified key regulatory mechanisms involving signaling pathways, oxidative stress, and antioxidant defense systems in the plant’s response to these stresses, offering crucial molecular insights into the complex stress responses of *D. huoshanense* (Figure 7). These findings not only enhance our understanding of plant responses to combined drought and high-temperature stress but also open new research avenues for identifying key genes and pathways involved in stress adaptation in *D. huoshanense*.

## Data Availability

The raw sequence data reported in this paper have been deposited in the Genome Sequence Archive (Genomics, Proteomics & Bioinformatics 2021) in the National Genomics Data Center (Nucleic Acids Res 2022), China National Center for Bioinformation/Beijing Institute of Genomics, Chinese Academy of Sciences (GSA: CR017872), and are publicly accessible at https://ngdc.cncb.ac.cn/gsa/ (accessed on 27 August 2024).

## Supplementary Materials

The following supporting information can be downloaded at: https://www.mdpi.com/article/10.3390/genes16030287/s1, Figure S1: Compositional analysis of differential metabolites and KEGG enrichment analysis of three comparison groups; Figure S2: QC sample UHPLC-OE-MS detection positive (right) and negative (left) ion mode TIC chart; Table S1: GO enrichment analysis of differentially expressed genes shared by theree groups; Table S2: The KEGG pathway of joint analysis shared by three groups; Table S3: Sequencing results of differentially expressed genes in three groups.

## Abbreviations

ROS	Reactive oxygen species
CAT	Catalase
SOD	Superoxide dismutase
POD	Peroxidase
MDA	Malondialdehyde
MAPK	Mitogen-activated protein kinase
