# Supplementary material for: Synergistic Regulation at Physiological, Transcriptional, and Metabolic Levels in Dendrobium huoshanense Plants Under Combined Drought and High-Temperature Stress
Source: Genes (Basel). 2025 Feb 27;16(3):287. doi: 10.3390/genes16030287 (PMC11942376; doi:10.3390/genes16030287)
Supplement: Supplementary file 1 [file genes-16-00287-s001.zip › Supplementary materials.pdf]

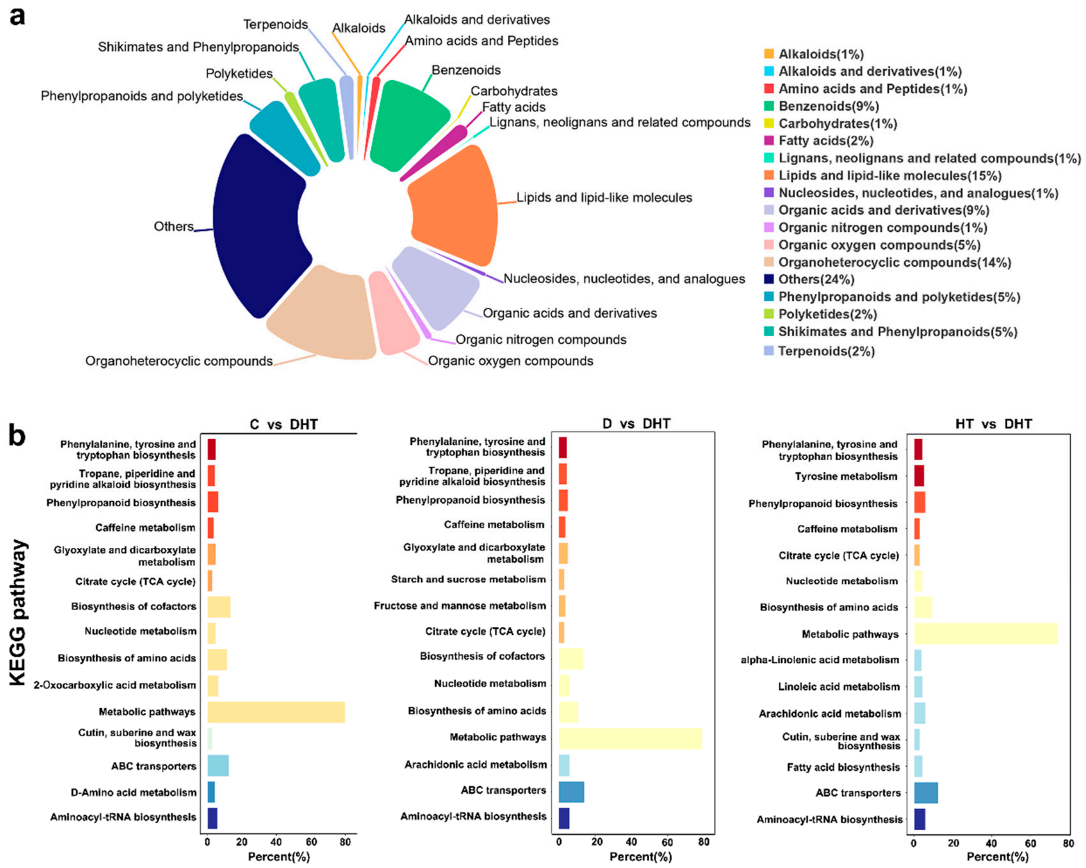

Figure S1. Compositional analysis of differential metabolites (a) and KEGG enrichment analysis of three comparison groups (b).

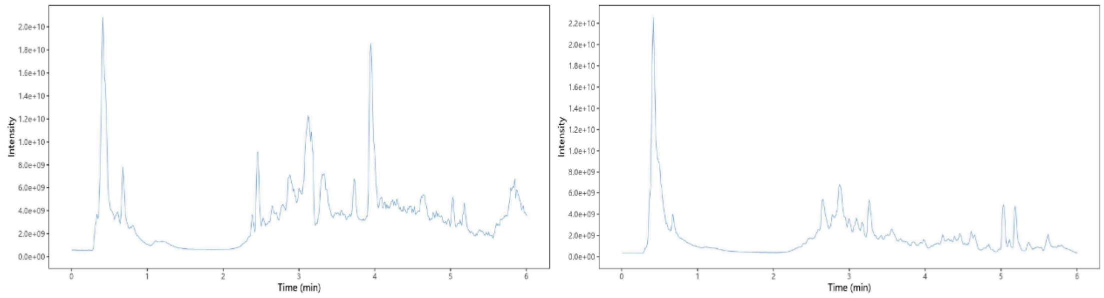

Figure S2. QC sample UHPLC-OE-MS detection positive (right) and negative (left) ion mode TIC chart.
